# PD39 - Application of population pharmacokinetic modeling and simulation in the design of the optimal dose regime of rupatadine in children 2-5 year old children

**DOI:** 10.1186/2045-7022-4-S1-P39

**Published:** 2014-02-28

**Authors:** Marta Valle, Javier Estevez, Lisa Charlotte Martial, Eva Santamaria, Iñaki Izquierdo

**Affiliations:** 1 Sant Pau, Barcelona, Spain; 2 Uriach, Barcelona, Spain

## Background

Rupatadine is a second generation antihistamine H1 and antagonist of PAF for the treatment of allergic rhinitis and urticaria for which a new pediatric oral solution is now available for children between 6-11 y/o.

## Objectives

1) To optimize the dose regime in children between 2 to 5 y/o old to reach similar plasma concentrations to children of 6-11 y/o with allergic rhinitis.

2) To build a new population pharmacokinetic (popPK) model in children including all ages (2-11 y/o) to evaluate if the proposed regimen, as a function of weight, is adequate to reach rupatadine exposure similar to adults and ≥ 12 y/o.

## Methods

A popPK model was developed, using data from 6-11 y/o study (STD I) including 11 patients with full PK profile in allergic rhinitis. A second study (STD2) including 2-5 y/o was optimal designed based on the parameters estimated from STD I, assuming: inclusion of < 40 children, < 5 samples per child in the shortest time window. A final popPK model was built for children 2-11 years. Influence of different covariates on model parameters was also evaluated. PopPK modeling and simulation was performed in NONMEM and optimal design in WINPOP software.

## Results

The dose administered in STD II was 2.5 mg/kg (weight 10-25 kg) or 5 mg/kg (weight > 25 kg) and 3 samples per child were needed in a 2h time window. A two-compartmental model with first-order absorption and elimination where clearance depends on weight fitted the data for 2-11 y/o children. Mean (SD) estimates of parameters obtained by noncompartmental analysis of the steady-state simulated plasma concentrations for both subsets of children were similar: Cmax, 2.54(1.26) vs 1.96(0.52) ng/mL; AUC, 10.74(3.09) vs 10.38(4.31) ng/mL/h; t1/2, 12.28(3.09) vs 15.94(4.09), for children 6-11 y/o and children 2-5 y/o, respectively.

## Conclusion

A popPK model for rupatadine was used in the design of a new clinical study. Rupatadine clearance in children 2-11 years increases with age. The used range of doses in children provides similar exposure to rupatadine to that associated with efficacy and safety in adults and adolescents ≥ 12 y/o.

